# COVID-19 Vaccine-Associated Sensorineural Hearing Loss and Tinnitus

**DOI:** 10.7759/cureus.93384

**Published:** 2025-09-28

**Authors:** András Szilágyi, Balázs Sztanó, Béla Juhász, Zoltán Szilvássy

**Affiliations:** 1 Department of Otolaryngology and Head and Neck Surgery, University of Debrecen, Debrecen, HUN; 2 Department of Pharmacology and Pharmacotherapy, Department of Emergency Clinic, University of Debrecen, Debrecen, HUN; 3 Department of Pharmacology and Pharmacotherapy, University of Debrecen, Debrecen, HUN

**Keywords:** sars-cov, sinopharm, snhl, tinnitus, vaccine

## Abstract

The COVID-19 pandemic has increased the incidence of otolaryngological symptoms, including tinnitus and sensorineural hearing loss (SNHL), following both infection and vaccination. Tinnitus remains a prevalent and difficult-to-treat symptom, with limited reported cases linked to the Sinopharm vaccine. We present the case of a 79-year-old female with hypertension who developed bilateral tinnitus seven days after her first Sinopharm COVID-19 vaccination and had previously recovered from COVID-19 without postinfection sequelae. Audiometry revealed high-frequency SNHL in both ears. Despite the vitamin B complex and multiple courses of piracetam, her hearing did not improve, although her tinnitus handicap inventory (THI) score slightly decreased. Tinnitus following COVID-19 vaccination is rare, with even fewer cases associated with inactivated vaccines such as Sinopharm. Management remains challenging, and the psychological impact of the pandemic may contribute to symptom persistence. Early intervention and awareness are essential for optimal care.

## Introduction

The COVID-19 pandemic has imposed a substantial burden on the healthcare system. Post-COVID syndrome can manifest with several otolaryngological symptoms, including tinnitus, vertigo, and olfactory dysfunction. Both SARS-CoV-2 infection and vaccination have been associated with the onset of tinnitus and sensorineural hearing loss (SNHL). Tinnitus is a prevalent yet challenging otological symptom to treat, and its reported prevalence in the literature ranges from 5.1% to 42.7% [1]. Although numerous treatment options for tinnitus are outlined in clinical guidelines, not all options are accessible to every clinician, and tinnitus symptoms remain difficult to manage in certain cases. Our review of the international literature identified 27 reported cases, with only one patient having received the Sinopharm vaccine prior to developing tinnitus and SNHL [2]. This patient, a 79-year-old female and the oldest in the case reports, presented to our outpatient clinic with new-onset tinnitus following her first vaccination.

## Case presentation

**Figure 1:**
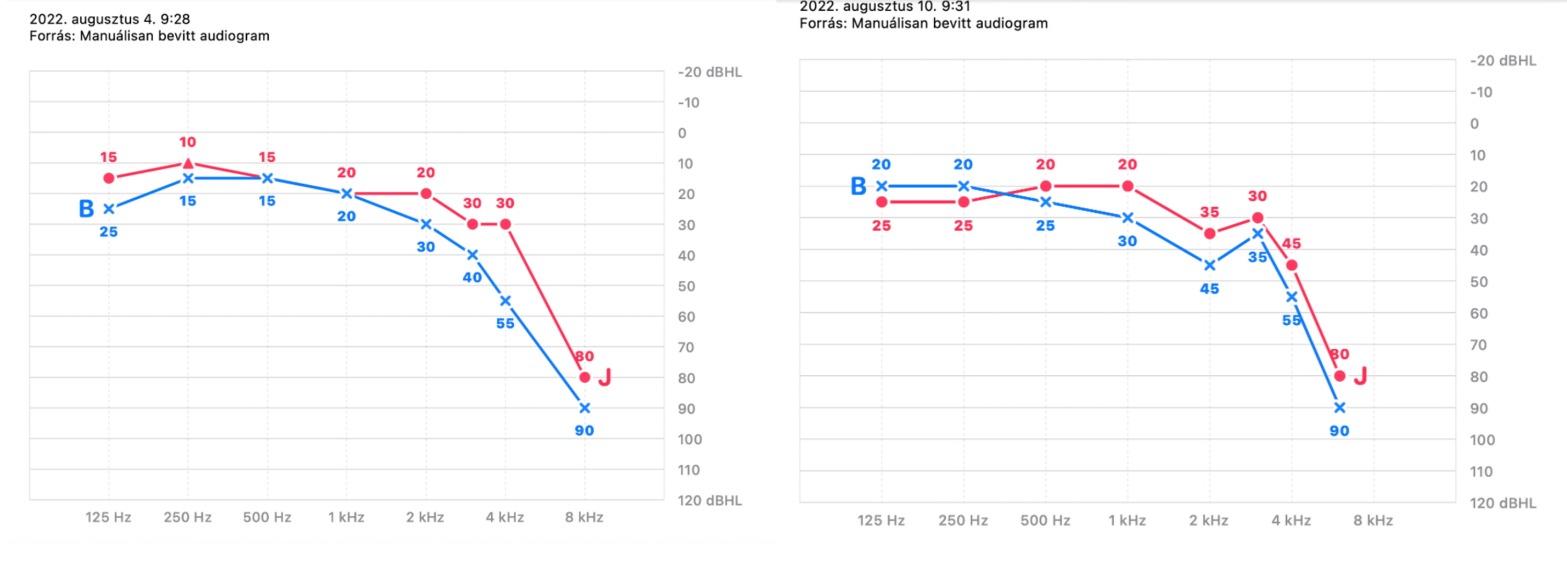
Audiogram before and after treatment As shown in the figure, no improvement in audiometric outcomes was observed following the treatment. A persistent high-frequency sensorineural hearing loss bilaterally was observed.

A 79-year-old female patient with a medical history limited to hypertension presented to our outpatient clinic with bilateral tinnitus, which manifested following her initial dose of the Sinopharm vaccine. The patient had previously contracted COVID-19 on November 4, 2020, which was confirmed via PCR, with initial symptoms including fever, myalgia, and weakness. Postinfection, there were no indications of post-COVID syndrome. She received her first dose of the Sinopharm vaccine on March 4, 2021, and developed tinnitus seven days later. Subjective audiometry conducted on April 29, 2022, indicated good hearing on the right side at low frequencies, mild hearing loss at 3000-4000 Hz, and sensorineural hearing loss of 80 dB at 8000 Hz. On the left side, hearing was good at 125-1000 Hz and moderate at 2000-4000 Hz, with sensorineural hearing loss of 90 dB observed at 8000 Hz. Her Tinnitus Handicap Inventory (THI) score was 45. Alternative etiologies of tinnitus were excluded on the basis of neurological examination and computed tomography. The patient commenced vitamin B complex supplementation, and upon her insistence on additional pharmacological intervention, we initiated a regimen of 6 grams of piracetam infusion over five days in August. A follow-up audiometric evaluation at the conclusion of rheological therapy revealed no improvement, prompting the initiation of oral piracetam (2 × 1200 mg) (Figure 1).

At her subsequent evaluation in January 2023, her hearing deteriorated. Another course of rheological treatment was administered in March; however, no improvement was observed in either her hearing or tinnitus, but her THI score improved to 38 points.

## Discussion

Tinnitus following COVID-19 vaccination is an infrequent but increasingly recognized phenomenon. Large-scale data confirm its rarity: in 2022, Dorney et al. analyzed 2.5 million patients and reported a prevalence of new-onset tinnitus of 0.038% after the first vaccination and 0.031% following the second dose, with higher rates observed after non-COVID vaccinations [3]. Similarly, Parrino et al. described three cases of post-vaccination tinnitus, of which two patients improved spontaneously or with treatment, while one declined therapeutic intervention [4]. In our literature review for this case report, including the presented case, the mean reported patient age was 51.37 years, with a female-to-male ratio of 1:2.85. Our patient represents one of the oldest documented cases and highlights the vulnerability of elderly patients to persistent auditory complications.

The mechanisms underlying tinnitus and SNHL after COVID-19 vaccination remain poorly understood. Proposed pathophysiological pathways include immune-mediated inner ear injury, molecular mimicry between viral antigens and cochlear proteins, microvascular disturbances, and neuroinflammatory processes comparable to those observed in other post-viral syndromes [5,6]. Given the cochlea’s high metabolic demand and limited vascular reserve, microcirculatory compromise may be particularly detrimental, especially in patients with comorbid vascular risk factors such as hypertension [7].

Tinnitus is also strongly influenced by psychosocial dimensions. Health-related anxiety, social isolation, and pandemic-associated psychological stress have all been shown to exacerbate tinnitus perception, potentially explaining symptom persistence even when objective hearing remains stable [8]. This underlines the importance of a holistic management approach in tinnitus care.

Regarding vaccine type, evidence suggests that inactivated vaccines, such as influenza or Tdap, are more often associated with tinnitus than mRNA or vector vaccines [3]. However, with COVID-19 vaccines, reports remain scarce. To date, only two known case reports, including ours, implicate the inactivated Sinopharm vaccine, making this association especially noteworthy [2]. Additional case accumulation is required to establish causality.

Treatment outcomes for post-vaccination tinnitus are heterogeneous. Corticosteroids are standard of care for sudden SNHL [9], but their efficacy in chronic tinnitus remains controversial. In our patient, the absence of prompt corticosteroid therapy may have contributed to the lack of auditory recovery. Instead, therapies such as vitamin B complex and piracetam provided only modest relief, reflected by the slight improvement in THI score from 45 to 38 points. While no audiometric improvement was achieved, partial symptom adaptation occurred, underscoring the importance of early therapeutic intervention, patient education, and psychological support.

Future studies involving larger cohorts and longitudinal follow-up are needed to clarify risk factors and pathogenic mechanisms. Until then, clinicians should remain vigilant for auditory complications following vaccination, particularly in elderly patients with vascular comorbidities. Equally important, communication with patients should stress both the extremely low risk of otological side effects and the overwhelming benefits of vaccination in reducing morbidity and mortality during the COVID-19 pandemic.

## Conclusions

COVID-19 vaccinations infrequently result in tinnitus. Despite the availability of numerous treatment options, tinnitus remains a challenging symptom to manage. Notably, as highlighted in Dorney et al.'s review, inactivated vaccines such as influenza or Tdap are more frequently associated with tinnitus. Nevertheless, concerning COVID-19 vaccines, only two cases have been reported in association with Sinopharm, which is also an inactivated vaccine. Furthermore, the psychological impact of the pandemic must be considered when discussing the etiology of tinnitus.
